# CD34+ hematopoietic progenitor cell dose as a predictor of engraftment and survival in multiple myeloma patients undergoing autologous stem cell transplantation

**DOI:** 10.3906/sag-2001-173

**Published:** 2020-12-17

**Authors:** Elifcan ALADAĞ, Haluk DEMİROĞLU, Yahya BÜYÜKAŞIK, Mehmet TURGUT, Salih AKSU, Nilgün SAYINALP, İbrahim Celalettin HAZNEDAROĞLU, Osman İlhami ÖZCEBE, Hakan GÖKER

**Affiliations:** 1 Department of Hematology, Faculty of Medicine, Hacettepe University, Ankara Turkey; 2 Department of Hematology, Faculty of Medicine, Ondokuz Mayıs University, Samsun Turkey

**Keywords:** Multiple myeloma, CD34+hematopoietic progenitor cell, engraftment, survival

## Abstract

**Background/aim:**

High-dose melphalan and autologous hematopoietic stem cell transplantation (AHSCT) is the standard treatment strategy for multiple myeloma (MM) patients who are eligible for it. The recommended dose of CD34+ hematopoietic progenitor cells (HPCs) for adequate engraftment is above 2 × 106/kg. The aim of this study was to evaluate the relationship between the dose of CD34+ HPCs and survival in MM patients who underwent AHSCT at a tertiary care center.

**Materials and methods:**

Enrolled in this study were 271 MM patients who underwent AHSCT between 2003 and 2019. Clinical characteristics of the patients, disease status pre-AHSCT, reinfused CD34+ cell doses, and neutrophil and platelet engraftment days were recorded, retrospectively. The patients were divided into 2 groups according to whether the dose of reinfused CD 34+ HPCs was <5 × 106/kg or ≥5 × 106/kg. The groups were compared in terms of engraftment and overall survival (OS) times.

**Results:**

The median age of the patients was 54.8 (33–76) years. The median dose of infused CD34+ HPCs was 5.94 × 106/kg (1.47–59.5 × 106/kg). The median follow-up period was 54 months (4–211). The median OS of the patients was 103 months (11–144). The median neutrophil and platelet engraftment time was 10 (8–24) and 11 (7–40) days. Doses of <5 × 106/kg and ≥5 × 106/kg CD34+ HPC were reinfused in 38.1% and 61.9% of the patients, respectively. There was a negative significant correlation between the reinfused CD34+ cell level and neutrophil/platelet engraftment times (r = –0.32, P < 0.001; r = –0.27, P < 0.001, respectively). The median OS times were observed as 103 months (11–144) and 145 months (123–166) for patients who had been administered <5 × 106/kg and ≥5 × 106/kg of CD34+ HPCs, respectively (P = 0.009).

**Conclusion:**

The increased amount of CD34+ autologous hematopoietic stem cell dose after high dose melphalan chemotherapy in MM patients shortened the platelet and neutrophil engraftment time and increased OS. Early platelet engraftment and administration of a CD34+ HPC count that is ≥5 × 106/kg can be considered as predictors of better survival in patients.

## 1. Introduction

Autologous hematopoietic stem cell transplantation (AHSCT) in eligible multiple myeloma (MM) patients is currently a standard treatment modality [1]. The amount of CD34+ hematopoietic progenitor cells (HPCs) that can be mobilized in AHSCT may vary depending on the age, dose, and duration of chemotherapies, and the bone marrow involvement of the disease [2]. The increase in the amount of reinfused stem cells reduces the risk of complications and hospitalization by shortening the neutrophil and platelet engraftment times [3,4]. Many studies have been conducted to determine the appropriate amount of stem cells to be administered. The infusion of CD34+ HPCs above 2 × 106/kg is usually the recommended dose to prevent engraftment failure or late recovery [5,6]. There are studies that have shown that administering <5 × 106/kg of CD34+ HPCs prolongs the engraftment time and, especially, adversely affects platelet recovery [7]. The International Myeloma Working Group (IMWG) recommends that an average of 8 × 106/kg CD34+ should be given if mobilized, and that the minimum administration target should be 4 × 106/kg CD34+ progenitor cells [8].

In the present study, the association between CD34+ HPC level, and engraftment and survival time in MM patients who underwent AHSCT at the Hacettepe University Hematology Department was analyzed.

## 2. Material and methods

### 2.1. Study population

This study included 271 MM patients who underwent AHSCT at the Hacettepe University Department of Hematology between 2003 and 2019. All of the data were analyzed retrospectively. As a standard of care/action of the hospitals of Hacettepe Medical School, all of the ethical considerations were strictly followed, all of the patients gave informed consent for the procedure at the time of hospitalization, and before the administration of chemotherapy and other relevant diagnostic/therapeutic procedures, in accordance with the Declaration of Helsinki.

### 2.2. Mobilization of peripheral blood stem cells

All of the patients received 4 ± 2 courses of bortezomib-dexamethasone, bortezomib-cyclophosphamide-dexamethasone, or vincristine-adriamycin-dexamethasone chemotherapy during the prebortezomib era, and 28 day schedules for induction chemotherapy for MM [9]. Granulocyte colony stimulating factor was administered at a dose of 10 µg/kg for a minimum of 5 days. When the CD34 level ≥20 µL, the patients underwent leukapheresis. Additional apheresis was performed with Plerixaphor in 24 patients, due to the insufficient amount of stem cells had been collected.

### 2.3. Response

The neutrophil engraftment time was accepted as the first of 3 consecutive days, with an observed absolute neutrophil count of ≥0.5 × 109/L. The platelet engraftment time was accepted as the first of 3 consecutive days with a platelet count that measured ≥20 × 109/L, without the need for replacement.

### 2.4. Endpoints

The primary aim of this study was to demonstrate the correlation between the CD34+ HPC level, and the platelet and neutrophil engraftment times. The secondary aim was to investigate the effect of the infused CD34+ HPC count at the 5 × 106/kg cut-off value on long-term overall survival (OS).

### 2.5. Statistics

Patient and transplant characteristics were summarized using descriptive statistics. The chi-square test was used for the statistical comparisons between the disease groups. The independent sample t test was used the compare the parameters if the data were normally distributed, while the Mann-Whitney U test was used for the nonnormally distributed data. Univariate linear regression analysis was performed for each potential risk variable. As independent variables, baseline demographic and clinical characteristics at P < 0.1 via univariate analysis were included into the multivariate regression analysis using the stepwise model to predict engraftment and survival. During the multivariate regression analysis, tolerance and variance inflation factor values of collinearity statistics were taken into consideration to avoid potential strong correlation among the independent variables.

All of the patients were followed longitudinally until death or last follow-up. Kaplan Meier curves were used to estimate the OS calculated from the time of diagnosis date until death, or to the last follow-up evaluation. The relationship between the platelet/neutrophil engraftment time and CD34+ HPC dose was evaluated using Spearmen correlation analysis. All statistical analyses were performed using IBM SPSS Statistics for Windows 25.0 (IBM Corp., Armonk, NY, USA).

## 3. Results

### 3.1. Patients characteristics

The median age of the patients included in the study was 54.8 (33–76) years. The median amount of infused CD34+ HPCs was 5.94 × 106/kg (1.47–59.5 × 106/kg). When the administered CD34+ HPC cut-off value was taken as 5 × 106/kg, 103 patients had stem cell infusion below this cut-off level, while 168 patients were above this level. The comparison of the clinical baseline characteristics of both groups are given in Table 1.

**Table 1:** Patient characteristics based on reinfused CD34+ HPC levels. CR: complete remission, VGPR: very good partial remission, PR: partial remission, F: female, M: male, Ig: immunoglobulin.

	Reinfused CD34+HPC dose (×106/kg)		
	<5	≥5	P value
n	103	168	
Sex (F/M)	31/72	70/98	0.037
Age	54.8	54.7	0.86
Type of MM			0.163
IgA kappa	17	24	
IgA lambda	10	11	
IgG kappa	30	66	
IgG lambda	25	24	
IgD kappa	1	1	
IgD lambda	1	3	
Kappa light chain	8	26	
Lambda light chain	11	13	
Disease status (pre-AHSCT)			0.17
CR	9	9	
VGPR	11	31	
PR	67	97	
Refractory	12	27	
Type of chemotherapy			0.14
VAD	70	94	
VD	15	33	
VCD	18	41	

VD: bortezomib-dexamethasone, VCD: bortezomib-cyclophosphamide-dexamethasone, VAD: vincristine-adriamycin-dexamethasone.

### 3.2. Hematologic recovery

**Figure 1:**
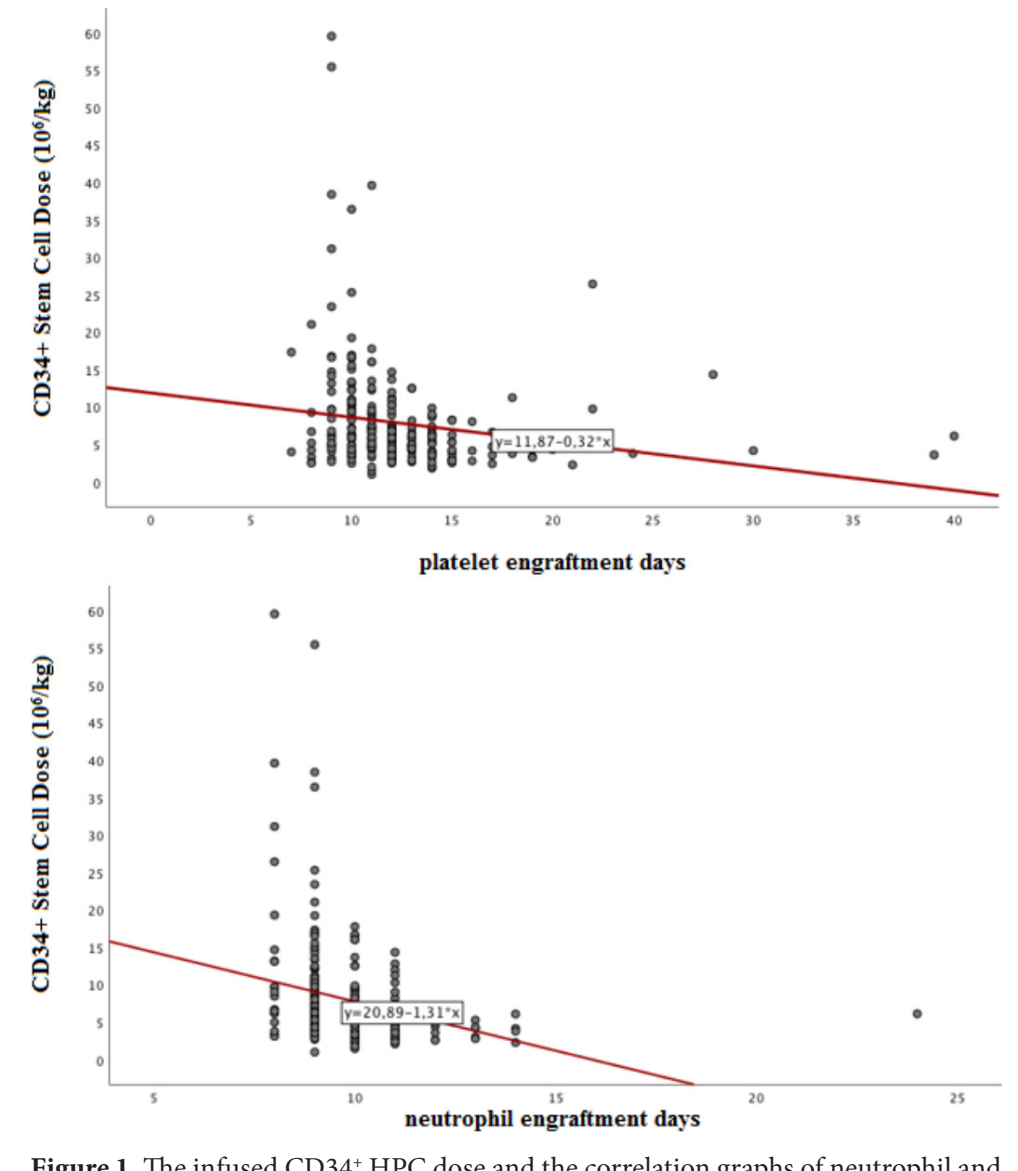
The infused CD34+ HPC dose and the correlation graphs of neutrophil and platelet engraftment times (r = –0.32, P < 0.001; r = –0.27, P < 0.001, respectively).

The median platelet engraftment time was 11 days (7–40) and the median neutrophil engraftment time was 10 days (8–24). Engraftment failure was not observed in any of the patients. When the neutrophil and platelet recoveries were evaluated, there was a significant negative correlation between the engraftment time and administered CD34+ HPCs in both groups (Figure 1).

### 3.3. Overall survival

The median follow-up period of the patients was 54 months (4–211). A total of 60 patients, who had been followed-up, died during this period. Of these patients, 61% had died due to infectious causes. The 100-day transplantation-related mortality was 1.1% (3 patients), mainly due to the viral infection (H1N1)-related mortality.

**Figure 2:**
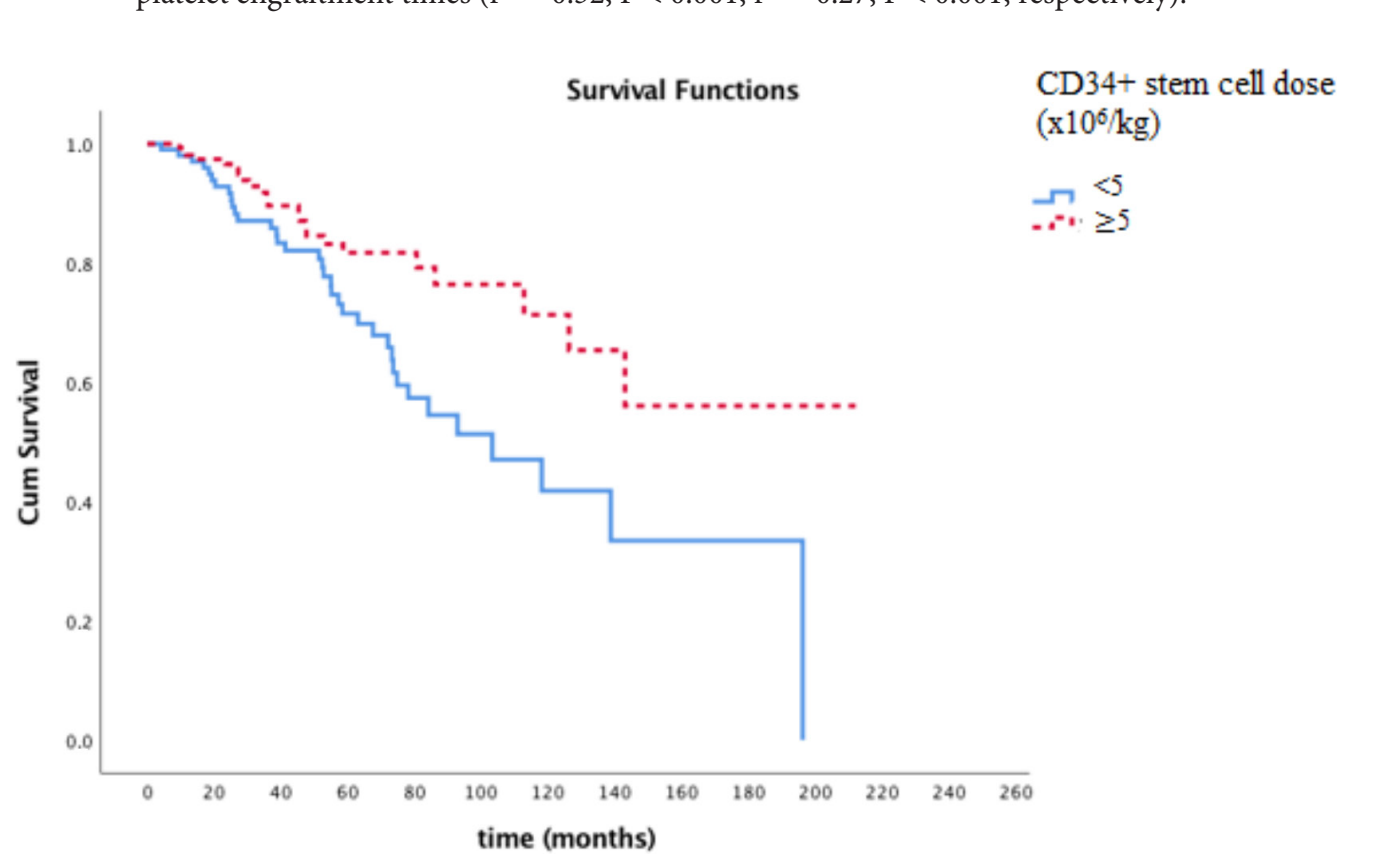
The Kaplan-Meier survival graph showing the difference in OS in patients with infusion of above and below 5 × 10 ^6^ /kg CD34+ HPC/kg (P = 0.009).

The median OS time was 103 months (11–144) for patients who were administered <5 × 106/kg of CD34+ HPCs and 145 months (123–166) for patients who were administered ≥5 × 106/kg of CD34+ HPCs (P = 0.009) (Figure 2). When evaluated using multivariate analysis, the CD34+ HPC level of <5 × 106/kg, and lambda subtype and platelet engraftment time above 10 days were observed to have a poor prognostic effect on OS (Table 2).

**Table 2:** Hazard ratio (HR) for clinical factors in the OS of MM patients who underwent AHSCT.

	Univariate		Multivariate	
	OS			
	HR	p	HR	P value
Age (<65, ≥65)	1.2 (1.1-1.4)	0.44		
Sex (F/M)	0.14	0.51		
Type of heavy chain				
IgA	1.1 (0.9–1.4)	0.55		
IgG	1.1 (0.9–2.13)	0.42		
IgD	1.5 (0.8–2.4)	0.053	2.8 (0.5–15)	0.22
Type of light chain (kappa/lambda)	1.12 (0.97–1.27)	0.056	0.6	0.039
ISS score (reference score1)				
2	1.18 (0.9–1.4)	0.67		
3	1.24 (1–1.4)	0.51		
Disease Status pre-AHSCT (reference CR)				
PR	1.5 (1.2–1.9)	0.52	0.5 (0.2–2.5)	0.529
Refracory	1.3 (1–1.7)	0.88		
Reinfused CD34+ HPC (<5 or ≥5 × 106/kg)	1.3 (1.2–1.4)	<0.001	1 (1.4–5.6)	0.021
Neutrophil engraftment time (<10, ≥10 days)	1.1 (0.9–1.2)	0.03	0,59	0.48
Platelet engraftment time (<10, ≥10 days)	1.11 (0.83–1.38)	0.027	0,9	0.029

## 4. Discussion

MM constitutes about 10% of hematologic malignancies and approximately 1% of all malignancies [10]. Currently, the standard treatment is high-dose melphalan with AHSCT for suitable patients [1]. The amount of CD34+ HPCs mobilized for reinfusion for AHSCT may vary depending on the type and duration of chemotherapy, age, disease status, and radiotherapy history of the individual. Collection of CD34+ HPCs below 1 × 106/kg is identified as mobilization failure, while the collection of CD34+ HPCs between 1–5 × 106/kg is identified as inadequate mobilization [11]. In a previous study, it was shown that adequate mobilization was achieved in 94% of patients receiving treatment for less than 2 years and in 28% of patients receiving treatment for a longer period of time [5]. It is known that the high amount of reinfused CD34+ HPCs reduces hospitalization by shortening the engraftment time [12,13]. However, although there is no clear consensus on the border CD34+ level, it has been stated by the MM IGWS group that the administration of CD34+ HPCs above 4 × 106/kg will be sufficient for a healthy engraftment period [14]. In many studies, it was found that a CD34 dose of 2.5 × 106/kg was sufficient for engraftment, while administering stem cells ≥5 × 106/kg has been shown to shorten the engraftment time. A limited number of studies have shown that administering CD34+ HPCs at a dose of ≥11 × 106/kg prolonged hospitalization time by causing fever and engraftment syndrome. In one study, engraftment was achieved on day 21 in 63% of the patients with 2.5–5 × 106/kg of the CD34+ stem cells collected, while a platelet value of ≥50 × 109/L was obtained in 93% of the patients administered above 5 × 106/kg of CD34+ HPCs. In the present study, the median platelet engraftment time was found as 10 days in all of the patients who had been engrafted adequately. In this study, a shorter platelet engraftment time was observed as an independent predictive factor for OS.

It was shown that the neutrophil engraftment time was 18, 15, and 13 days in 95% of the patients receiving CD34+ HPCs at cut-off values of 2.5, 5, and 7.5 × 106/kg, respectively [15].

In a different study, it was shown that the administration of 2.5–5 × 106/kg or 5 × 106/kg CD34+ HPCs did not provide any difference in survival (P = 0.186) [5]. In the current study, a significant negative correlation was observed between the neutrophil engraftment time and the number of CD34+ HPCs. When multivariate analysis was performed, it was shown that it was not predictive of OS.

Additionally, the multivariate analysis for investigating the predictive factors for survival showed that a low CD34 level, platelet engraftment time of ≥10 days, and lambda light chain types were poor prognostic factors. There have been clinical studies showing that patients with high serum lambda levels had low survival rates. According to one clinical study, the 5-year survival rate was 63% in patients with free lambda levels below the median value and 37% in patients above the median value (hazard ratio = 2.44, 95% CI: 1.50–3.97, P = 0.001) [16]. The lambda subtype was shown to be a poor prognostic factor in the study, but no analysis in accordance with this finding was performed for lambda light chain levels [16].

In the present study, the CD34+ HPC dose above the 5 × 106/kg threshold was demonstrated to positively impact OS. This was attributed to the fact that the mobilized CD34+ HPC levels of the patients were possibly associated with the bone marrow reserves, and that patients with low bone marrow reserves were somewhat susceptible to infection. In this study, 61% of the deceased patients had died due to infectious causes, mainly comprising encapsulated bacterial and viral infections, such as influenza.

## 5. Conclusion

The increased dose of CD34+ autologous hematopoietic stem cells after high-dose melphalan chemotherapy in MM patients shortened the platelet and neutrophil engraftment time and increased OS. Early platelet engraftment and the administration of a CD34+ HPC count that is ≥5 × 106/ kg can be considered as predictors of a better survival in patients. However, further larger multicentered studies are needed to better elucidate the ideal CD34+ HPC dose affecting OS in MM.

## Informed consent

As a standard of care/action of the hospitals of Hacettepe Medical School, all of the ethical considerations were strictly followed, all of the patients gave informed consent for the procedure at the time of hospitalization and before the administration of chemotherapy and other relevant diagnostic/therapeutic procedures were followed in accordance with the Declaration of Helsinki.
